# Antimicrobial resistance profile and presence of class I integrongs among *Salmonella enterica* serovars isolated from human clinical specimens in Tehran, Iran

**Published:** 2011-09

**Authors:** F Firoozeh, F Shahcheraghi, T Zahraei Salehi, V Karimi, MM Aslani

**Affiliations:** 1 Department of Microbiology, Faculty of Veterinary Medicine, University of Tehran, Tehran, Iran.; 2 Department of Microbiology and Mcrobiology Research Center Pasteur Institute, Tehran, Iran.; 3 Clinical Sciences Department, Avian Diseases Section, Faculty of Veterinary Medicine, University of Tehran, Tehran, Iran.

**Keywords:** Antibiotic resistance, class 1, integrons, *Salmonella*, serovars

## Abstract

**Background and objectives:**

*Salmonella* is one of the leading causes of food-borne diseases. Increasing occurrence of antimicrobial resistance, especially multidrug-resistance, in *Salmonella* serovars is a major public health problem worldwide. This study was carried out to detect class I integrons and antibiotic resistance profiles in clinical isolates of *Salmonella* serovars collected from seven hospitals in Tehran during November 2009 to June 2010.

**Materials and Methods:**

Antibiotic susceptibility profile of 19 antibiotics against 58 *Salmonella* isolates commonly used in humans was determined using disk diffusion assay. Minimum inhibitory concentration against ceftriaxone and ciprofloxacin was studied. PCR assays were used to detect class I integrons.

**Results:**

Among 58 *Salmonella* isolates, 72.4% were *Salmonella enterica* serovar Enteritidis, 8.7% were *Salmonella* enterica serovar Typhimurium and 18.9% were other serovars. Of the total 58 *Salmonella* serovars, 43 (74.1%) were multidrug-resistant and showed resistance to three or more antibiotic families. Class I integrons were identified in 38 (88.3%) MDR *Salmonella* isolates. Ciprofloxacin minimum inhibitory concentration ranged between 0.125-2 g/ml for four isolates and other four isolates exhibited resistance to ceftriaxone (MIC 64-256 g /ml).

**Conclusion:**

The high prevalence of class I integrons was seen in our MDR *Salmonella* isolates and class I integrons might play an important role in the dissemination of antimicrobial resistance determinants.

## INTRODUCTION

Food borne disease caused by non-typhoid *Salmonella* are found to be a major public health problem worldwide (1). Intestinal salmonellosis is self limiting; however, it may lead to systemic symptoms in children, the elderly and immunocompromised cases (2). Bacteremia is reported to occur in 3 to 10 percent of cases and in such situations, antimicrobial therapy is lifesaving (1). Increasing emergence of antibiotic resistance, especially multidrug-resistance, in *Salmonella enterica* is an important public health concern. Antimicrobial resistance genes may be spread via mobile genetic elements such as plasmids, transposons and integrons (3). Integrons are genetic elements that recognize and capture mobile gene cassettes, which usually encode antimicrobial drug resistance determinants. Integrons are usually found in association with transposons and are often located on plasmids, facilitating their mobility. Integrons are thus ideally suited for the dissemination and recombination of antimicrobial drug-resistance genes (4). Strong association between multidrug-resistant (MDR) *Salmonella* and the presence of integrons especially class I integrons, has been documented (4, 5). Class I integrons are the most common integrons found in clinical isolates of *Salmonella enterica* . Class I integrons consist of a 5'-conserved segment including the integrase gene *(int1)* and a 3'-conserved segment including the *qacE*Δ and *sul1* genes, conferring resistance to quaternary ammonium compounds and sulfonamides, respectively. The two conserved segments are separated by a variable region that usually contains one or more resistance gene cassettes (4).

The data concerning class I integrons resistance genes are limited in Iran (6). Therefore, the aim of the current study was to investigate the presence of class I integrons and antibiotic resistance profile of *Salmonella enterica* serovars isolated from clinical specimens.

## MATERIALS AND METHODS

*Salmonella* isolates. A total of 58 *Salmonella* isolates were investigated. Thirty-eight isolates were collected from seven hospitals in Tehran (Milad hospital, n = 15; Imam Khomeini, n = 7; Bahrami, n = 4; Sharyati, n = 3; Aliasghar, n = 4; Taleghani, n = 3 and Sina, n = 2) during November 2009 to June 2010. In addition, 20 human *Salmonella* isolates were obtained from University of Tehran. They were collected during the same period from clinical samples were referred to the Department of Microbiology, Faculty of Veterinary Medicine, University of Tehran.

Identification of isolates as *Salmonella* was confirmed by using conventional standard biochemical and serological tests (7). Multiplex PCR was used for serotyping of isolates to identify *Salmonella enterica* serovars Enteritidis ( *S.* Enteritidis) and Typhimurium ( *S.* Typhimurium) as described previously (8). Amplification was carried out in a Techne TC-512 thermocycler (Techne, UK) as follows: 35 cycles of 30 s for denaturation at 94 °C, 90 s for annealing at 56°C, and 30 s for primer extension at 72°C, followed by a terminal extension at 72°C for 10 min in the case of *S.* Enteritidis. Target genes for *S.* Typhimurium were amplified using the same thermocycler, as follows: 30 cycles of denaturation at 95°C for 1 min, annealing at 65°C for 1 min, primer extension at 72°C for 30 s, followed by 7 min at 72°C for terminal extension. For both amplifications, initial denaturation at 95°C for 5 min was used. Electrophoresis of PCR products were performed on 1.2% and 1.8% agarose gel for *S.* Typhimurium and *S.* Enteritidis isolates, respectively. The gels were stained in ethidium bromide for 15 minutes and visualized in gel document system (Biorad, UK). The primers used in this study are detailed in Tables 1 and 2. Remaining isolates that were negative in Multiplex PCR serotyping assays, were serotyped using commercial antisera (Difco, USA).

**Table 1:** Primers used for the detection of *Salmonella* Enteritidis (8).

Primer	Target gene	Length	Sequence (5’-3’)	Amplification Product (bp)
ST11	Random sequence *a*	24	GCCAACCATTGCTAAATTGGCGCA	429
ST14	Random sequence	25	GGTAGAAATTCCCAGCGGGTACTGG	
S1	*Spv**b*	20	GCCGTAGATACACGAGCTTA	250
S4	*spv*	20	ACCTACAGGGGCACAATAAC	
SEFA2	*sefA**c*	20	GCAGCGGTTACTATTGCAGC	310
SEFA4	*sef*	20	TGTGACAGGGACATTTAGCG	

a Randomly cloned sequence specific for the genus *Salmonella*

b *Salmonella* plasmid virulent gene

c *Salmonella* Enteritidis fimbrial antigen gen

**Table 2:** Primers used for the detection of *Salmonella* Typhimurium (8).

Primer	Target gene	Length	Sequence (5’-3’)	Amplification Product (bp
ST139-s	*inv* A	26	GTGAAATTATCGCCACGTTCGGGCAA	284
ST141-as	*inv* A	22	TCATCGCACCGTCAAAGGAACC	
Rfbj-s	*rfbJ*	24	CCAGCACCAGTTCCAACTTGATAC	663
Rfbj-as	*rfb* J	24	GGCTTCCGGCTTTATTGGTAAGCA	
Flic-s	*fli* C	23	ATAGCCATCTTACCAGTTCCCCC	183
Flic-as	*fli* C	24	GCTGCAACTGTTACAGGATATGCC	
Fljb-s	*flj* B	24	ACGAATGGTACGGCTTCTGTAACC	526
Fljb-as	*flj* B	24	TACCGTCGATAGTAACGACTTCGG	

Antimicrobial susceptibility testing. Antimicro-bial susceptibility testing was determined by the disk agar diffusion method according to Clinical and Laboratory Standards Institute (CLSI 2007) (9). Agar diffusion assays were performed on Muller Hinton agar. The following antimicrobial drugs: ampicillin (AMP: 10 g), gentamicin (GEN: 10 g), kanamycin (KAN: 30 g), streptomycin (STR: 10 g), chloramphenicol (CHL: 30 g), trimethoprim-sulfamethoxazole (SXT: 25 g), amoxicillin-clavulanic acid (AMC: 20/10 g), cefalothin (CEF: 30 g), cefotaxime (CTX: 30 g), ceftazidime (CAZ: 30 g), ceftriaxone (CRO: 30 g), aztreonam (ATM: 30 g), imipenem (IMP: 10 g), nalidixic acid (NAL: 30 g), ciprofloxacin (CIP: 5 g), norfloxacin (NOR: 10 g), doxycycline (DXT: 30 g), oxytetracycline (OT: 30 g), cefixime (CFM: 5 g), were used for antimicrobial susceptibility testing. The quality control organism *was Escherichia coli* ATCC 25922. Results were interpreted as susceptible or resistance according to criteria recommended by the CLSI and the manufacture protocols (BBL and Mast Companies, UK) (9). Intermediate isolates were counted as resistant.

Minimum inhibitory concentration. Minimum inhibitory concentrations (MICs) were determined by micro broth dilution method according to CLSI guidelines (9). The antimicrobials tested were ciprofloxacin and ceftriaxone (drugs of choice for treatment of systemic salmonellosis). The quality control organisms were *Escherichia coli* ATCC 25922 and *Pseudomonas aeroginosa* ATCC 27853

DNA extraction and integron class I detection. DNA from *Salmonella* isolates showing resistance to more than one antibiotic families were extracted using a standard methods (10). The integrase gene *(intI)* was amplified in PCR reaction for detection of class I integron. PCR assay was used to detect integron class I and the integrase *gene (intI)* was amplified. The primers were used for amplification of *intI* were: *intI* -F: GCCTTGCTGTTCTTCTACGG and *IntI* -R: GATGCCTGCTTGTTCTACGG (11).

Amplification was performed in a total volume of 25 l (24 l of PCR master mix and 1 l of extracted DNA as template) and carried out in a Thermo cycler (Eppendorf master cycler®, MA) using following cycling program: initial denaturation at 94°C for 5 min and 35 cycles of 30 s at 94°C, 30 s at 55-60°C and 2 min at 72°C, with a final extension for 5 min at 72°C (6). Reaction products were separated by gel electrophoresis and stained with ethidium bromide for visualization.

## RESULTS

In this study, 42 (72.4%) of 58 *Salmonella* strains identified as *Salmonella enterica* serovar Enteritidis, as well as 5 (8.6%) of the isolates belong to serovar Typhimurium based on multiplex PCR serotyping. Remaining isolates belonged to other serotypes ( *Salmonella* Paratyphi B, n = 5; *Salmonella* Paratyphi A, n = 4; *Salmonella* Paratyphi C, n = 1; *Salmonella* Havana, n = 1) based on serotyping with commercial antisera. The resistance patterns of *Salmonella* isolates to 19 antimicrobial agents are illustrated in Table 3. Of the 58 *Salmonella* serovars, 9 (15.5%) were susceptible to all antimicrobials tested and 43 (74.1 %) were multidrug-resistant and showed resistance to more than two antimicrobial families. Resistance pattern of isolates were also investigated and all *Salmonella* isolates could be grouped into 14 resistance phenotypes (Table 4).

**Table 3:** Antimicrobial susceptibility pattern of *Salmonella* isolates was determined by disk diffusion assay.

Antibiotic (s) tested *a*	Sensitive		Resistant	
	(No.)	(%)	(No.)	(%)
AMC	55	94.8	3	5.2
AMP	45	77.6	13	22.4
ATM	53	91.4	5	8.6
CAZ	51	87.9	7	12.1
CEF	57	98.2	1	1.8
CFM	54	93.1	4	6.9
CHL	48	82.2	10	17.2
CIP	57	98.2	1	1.8
CRO	54	93.1	3	6.9
CTX	56	96.6	2	3.4
DTX	13	22.4	45	77.5
GEN	54	93.1	4	6.9
IMP	58	100.0	0	0.0
KAN	45	77.6	13	22.4
NAL	15	25.9	43	74.1
NOR	57	98.2	1	1.8
OT	21	36.2	37	63.8
STR	19	32.7	39	67.3
SXT	46	79.4	12	20.6

a AMC (20/10 µg), Amoxicillin-clavulanic acid; AMP (10 µg), Ampicillin; ATM (30 µg),Aztreonam; CAZ (30 µg), Ceftazidime; CEF (30 µg), Cefalothin; CFM (5 µg), Cefixime; CHL (30 µg), Chloramphenicol; CIP (5 µg), Ciprofloxacin; CRO (30 µg), Ceftriaxone; CTX (30 µg), Cefotaxime; DTX (30 µg), Doxycycline; GEN (10 µg), Gentamicin; IMP (10 µg), Imipenem; KAN (30 µg), Kanamycin; NAL (30 µg), Nalidixic acid; NOR (10 µg), Norfloxacin; OT (30 µg), Oxytetracycline; STR (10 µg), Streptomycin; SXT (25 µg), Trimethoprim- Sulfamethoxazole.

**Table 4:** List of multidrug-resistant *Salmonella* isolates showing their antibiotic resistance phenotypes determined by disk diffusion method.

Antimicrobial resistance phenotype	No. of resistant * S * . enteritidis	No. of resistant * S * . Typhimurium	No. of resistant other Salmonella serovars	Total No. of resistant isolates
OT, STR	2	-	-	2
DTX NAL, STR	9	-	-	9
DTX, NAL OT	6	-	-	6
DTX, NAL OT, STR,	6	-	3 (Paratyphi A)	9
AMP, DTX, KAN, NAL OT, STR,	5	-	1 (Paratyphi A)	6
DTX, GEN NAL, OT, STR, SXT	1	-	-	1
DTX, KAN, NAL , OT, STR, SXT	-	1	-	1
ATM, AMP, CAZ, DTX,NAL, STR, SXT	-	-	1 (Havana)	1
AMC, CHL, DTX, KAN, NAL, OT, STR	1	-	-	1
AMP,ATM, CAZ, CHL, DTX, NAL, OT, STR, SXT	1	-	-	1
AMP, CAZ, CHL, DTX, KAN, NAL, OT, STR, SXT	-	3	1 (Paratyphi B)	4
AMC, CFM, CHL, CRO, DTX, GEN, NAL, OT, STR, SXT	1	-	1 (Paratyphi C)	2
AMP, ATM, CAZ, CFM, CHL,CTX, DTX, GEN, KAN, NAL, OT, STR, SXT	-	-	1 (Paratyphi B)	1
ATM, CEF, CFM, CHL CIP, CRO, CTX, DTX, NAL, NOR, OT, STR, SXT	1	-	-	1

AMC (20/10 µg), Amoxicillin-clavulanic acid; AMP (10 µg), Ampicillin; ATM (30 µg), Aztreonam; CAZ (30 µg), Ceftazidime; CEF (30 µg), Cefalothin; CFM (5 µg), Cefixime; CHL (30 µg), Chloramphenicol; CIP (5 µg), Ciprofloxacin; CRO (30 µg), Ceftriaxone; CTX (30 µg), Cefotaxime; DTX (30 µg), Doxycycline; GEN (10 µg), Gentamicin; IMP (10 µg), Imipenem; KAN (30 µg), Kanamycin; NAL (30 µg), Nalidixic acid; NOR (10 µg), Norfloxacin; OT (30 µg), Oxytetracycline; STR (10 µg), treptomycin; SXT (25 µg), Trimethoprim- Sulfamethoxazole.

One isolate demonstrated resistance to ciprofloxacin and norfloxacin, although 4 isolates showed decreased zone diameter for ciprofloxacin with MIC value ranging between 0.125-2 g/ml. The ceftriaxone MIC values were 64-256 g/ml for 4 of isolates that showed resistance to ceftriaxone in disk diffusion test. All strains were susceptible to imipenem and 6 isolates showed resistance to extended spectrum –lactam antibiotics like cefixime, ceftriaxone, aztreonam, and cefotaxime(Tables 3 and 4).

**Fig. 1:**
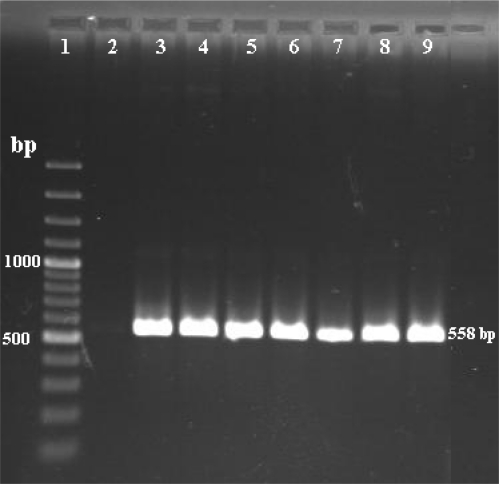
PCR amplification of class I integron *int1* gene in MDR *Salmonella* isolates. Lane 1: 1 kbp DNA ladder as the molecular size marker; lane 2: PCR mix with no template; lane 3: positive control; lanes 4-9 positive isolates

Of 43 MDR *Salmonella* isolates, 38 (88.3%) carried a class I integrase gene ( *intI* ) and possessed class I integrons (Fig. 1). Remaining isolates that were either susceptible to all antibiotics or resistant to at least two tested antibiotics, probably lacked class I integron. The presence of integrons was highly associated with resistance to 6-7 drugs including ampicillin, chloramphenicol, kanamycin, nalidixic acid, streptomycin, trimethoprim-sulfamethoxazole, and tetracyclines as 51.3% of isolates that carried class I integrons showed this multidrug-resistant pattern.

## DISCUSSION

Increasing antimicrobial resistance in *Salmonella* serovars is a major public health problem. This study shows the antibiotic resistance phenotypes and presence of class I integrons in clinical isolates of *Salmonella* serovars isolated in seven hospitals in Iran and Microbiology Department in Faculty of Veterinary Medicine, University of Tehran.

*Salmonella enterica* serovars Enteritidis and Typhimurium were reported to be the two most frequent serotypes of *Salmonella* isolated in Iran and other countries (12–16). Our findings show that the most prevalent isolated *Salmonella* serovars were *Salmonella* Enteritidis, which is in agreement with other reports (12–14).

When the resistance rate of our isolates was compared with previous studies in Iran, the resistance rates for our isolates were higher (12, 17). These results might be due to inadequate use of antimicrobial drugs in different fields and the spread of resistance determinants. Fluoroquinolone resistance in gram negative bacteria has been reported all over the world (3). Ciprofloxacin is a commonly prescribed fluoroquinolone in Iran. In this study, we showed resistance to ciprofloxacin and norfloxacin in one isolate (MIC 4 g/ml) with a reduced susceptibility to fluoroquinolone antibiotics in 4 isolates with a MIC values ranging between 0.125-2 g/ml. Previous reports indicated all *Salmonella* isolates were susceptible to ciprofloxacin (1, 12, 15, 17). Reports of resistance to ciprofloxacin are valuable data. Our study also shows emerging resistance of *Salmonella* isolates to extended spectrum –lactam antibiotics such as cefotaxime, ceftazidime, and ceftriaxone. These findings are of clinical significance because extended spectrum cephalosporins and fluoroquinolones are now the drugs of choice for treatment of invasive *Salmonella* infections in human (3).

Carbapenems (such as imipenem) are the main class of drugs used for treatment of infections caused by MDR and extended spectrum –lactamase producer gram negative bacteria including *Salmonella* (16, 18). In this study, like previous reports, all *Salmonella* isolates were susceptible to imipenem (12, 15, 17). This result is due to restricted prescription of carbapenems in Iran.

Our study showed that 74.1% (43/58) of *Salmonella* isolates have a multidrug-resistant phenotype. The resistance rate in this study was higher than a previous report by Naghoni *et al*. 2010 and indicates that antibiotic resistance in *Salmonella* serovars is an increasing problem in public health.

In the present study, 73.8% of *Salmonella enterica* serovar Enteritidis, 80% of *Salmonella enterica* serovar Typhimurium, and 72.7% of other *Salmonella* serovar isolates exhibited multidrug-resistance phenotype and showed resistance to 3-13 of tested antimicrobials. The most resistance was seen to ampicillin, chloramphenicol, kanamycin, nalidixic acid, streptomycin, trimethoprim-sulfamethoxazole, and tetracyclines. Similar resistance phenotypes have been previously reported in *Salmonella enterica* serovar Typhimurium and *Salmonella enterica* serovar Enteritidis isolates in Japan, France, Netherlands and Iran (16, 17, 19, 20). Identification of MDR isolates of *Salmonella enterica* serovars Typhimurium and Enteritidis are of great public health significance as these *Salmonella* serovars are two main causes of food borne salmonellosis in humans (21). Class I integrons were identified in 38 (65.5%) of *Salmonella enterica* isolates. We found that all integron-positive *salmonella* serovars were multidrug-resistant and intergron-negative as well as integron-positive isolates showed resistance to tetracyclines and streptomycin. These findings show that the class I integrons tested do not support the total resistance phenotypes observed among our *salmonella* isolates. Such results may be due to the presence of other integron classes or other genetic elements like transposons. The present survey shows 88.3% of the MDR *Salmonella* isolates carried class I integrons, which is indicative of high frequency of occurrence in MDR *Salmonella* serovars. The strong association between MDR *Salmonella* and presence of integron class I has been documented (4, 5). Here we report the widespread prevalence of integrons in *Salmonella enterica* serovars.

In conclusion, the high prevalence of integron–positive strains in our MDR *Salmonella* isolates indicates that these mobile genetic elements are common among different *Salmonella enterica* serovars and associate with reduced susceptibility to the first-line antimicrobial drugs.
